# MoleMCL: a multi-level contrastive learning framework for molecular pre-training

**DOI:** 10.1093/bioinformatics/btae164

**Published:** 2024-03-26

**Authors:** Xinyi Zhang, Yanni Xu, Changzhi Jiang, Lian Shen, Xiangrong Liu

**Affiliations:** Department of Computer Science and Technology, Xiamen University, Xiamen 361005, China; Department of Computer Science and Technology, Xiamen University, Xiamen 361005, China; Department of Computer Science and Technology, Xiamen University, Xiamen 361005, China; Department of Computer Science and Technology, Xiamen University, Xiamen 361005, China; Department of Computer Science and Technology, Xiamen University, Xiamen 361005, China; National Institute for Data Science in Health and Medicine, Xiamen University, Xiamen 361005, China

## Abstract

**Motivation:**

Molecular representation learning plays an indispensable role in crucial tasks such as property prediction and drug design. Despite the notable achievements of molecular pre-training models, current methods often fail to capture both the structural and feature semantics of molecular graphs. Moreover, while graph contrastive learning has unveiled new prospects, existing augmentation techniques often struggle to retain their core semantics. To overcome these limitations, we propose a gradient-compensated encoder parameter perturbation approach, ensuring efficient and stable feature augmentation. By merging enhancement strategies grounded in attribute masking and parameter perturbation, we introduce MoleMCL, a new MOLEcular pre-training model based on multi-level contrastive learning.

**Results:**

Experimental results demonstrate that MoleMCL adeptly dissects the structure and feature semantics of molecular graphs, surpassing current state-of-the-art models in molecular prediction tasks, paving a novel avenue for molecular modeling.

**Availability and implementation:**

The code and data underlying this work are available in GitHub at https://github.com/BioSequenceAnalysis/MoleMCL.

## 1 Introduction

In the current landscape of chemistry and biology research, efficiently deriving molecular representations is a pivotal challenge for crucial tasks, spanning from property prediction to drug design (Huang and Von Lilienfeld 2016). With the rise of deep learning across various domains, researchers are increasingly exploring the use of deep neural networks to achieve more precise molecular modeling (Jiménez-Luna *et al.* 2020, Pandey *et al.* 2022). In the domain of biological sequence analysis, some biological language models excel in efficiently and accurately handling DNA, RNA, and protein sequences (Li *et al.* 2021, Li and Liu 2023). However, leveraging molecular graphs, which encompass richer chemical features than SMILES sequences, allows models to better assimilate chemical knowledge (Hu *et al.* 2023). Accumulated research has adopted graph pre-training strategies (Qiu *et al.* 2020) to learn the molecule graph structure representation, i.e. molecular pre-trained models (MPMs). These models first undergo pre-training on a vast corpus of unlabeled molecular data, followed by fine-tuning to precisely address specific downstream tasks. This approach taps into the valuable information embedded in unlabeled data, laying a robust foundation for more intricate and specialized tasks.

As the current state-of-the-art (SOTA) method in unsupervised representation learning (He *et al.* 2020, Grill *et al.* 2020), the utilization of contrastive learning in graph structures has provided valuable perspectives for the pre-training of molecules (Sun *et al.* 2020). The essence of contrastive learning is to generate two views of the same sample through data augmentation and then maximize the similarity between their encoded representations (Gong *et al.* 2023). On the one hand, present methods primarily focus on structure-based augmentation techniques, such as randomly dropping or perturbing molecular components (nodes/edges) (You *et al.* 2020), or sampling important substructures of the molecule (Sun *et al.* 2020). However, these purely structure-based strategies fail to grasp the profound patterns of molecules as they operate on a simplistic assumption: molecules with similar structures should exhibit similar properties. For instance, considering molecular activity cliffs (Stumpfe *et al.* 2019), molecules with similar structures can exhibit entirely disparate characteristics, making it inappropriate to treat them as positive pairs in contrastive learning. Additionally, it is challenging to retain semantics during the current augmentation processes. Existing solutions select appropriate augmentations through manual trial and error, laborious optimization (You *et al.* 2020), or guidance from costly domain knowledge (Sun *et al.* 2021), yet these methods are often not the most ideal choices to preserve the complete semantics of molecules structures. Thus, Xia *et al.* (2022) proposed to implement perturbing model parameters rather than directly enhancing graph inputs, with the aim of profoundly learning the feature-level semantics of graph data. However, this method has its limitation: the noise extracted from simple distributions is random, which challenges the accurate capture of the intrinsic relationship between encoder parameters and graph embedding outputs (Gong *et al.* 2023)

Therefore, our goal is to devise a more scientifically grounded perturbation strategy to accurately and comprehensively capture the semantic features of molecular graphs. Inspired by the gradient reversal strategy in domain adaptation (Ganin and Lempitsky 2015), we introduce a gradient-compensated parameter perturbation scheme. Traditional gradient-based approaches, such as adversarial perturbations (Goodfellow *et al.* 2015, Miyato *et al.* 2017, Madry *et al.* 2018), require labeled supervised training, which poses a significant challenge due to the scarcity of labeled data in molecular research. To address this, we employ the previously established attribute masking technique (Hu *et al.* 2020), which facilitates the initial extraction of structural-semantic insights. Building on this, we implement contrastive learning with attribute masking, providing computable gradients for perturbations in encoder parameters. Thus, by integrating both structure-level and feature-level graph contrastive learning, we introduce a new MPM, MoleMCL.

The primary contributions of this article are as follows:

1. As a countermeasure, we introduce a new contrastive learning strategy: a gradient compensation scheme for encoder parameter perturbation. This method perturbs model parameters in a more scientific manner, ensuring stability and effectiveness during the contrastive learning process.
2. To capture the structural semantics and feature semantics of molecular graphs concurrently, we combine attribute masking and encoder perturbation-based augmentations. This integration leads to the introduction of MoleMCL, a multi-level contrastive learning framework for molecular pre-training.
3. Experimental evaluations reveal that MoleMCL either matches or outperforms current SOTA models in molecular property prediction tasks. Furthermore, the two augmentation techniques can be utilized as foundational pre-training tasks, achieving benefits across various graph neural networks (GNNs) architectures.

## 2 Materials and methods

### 2.1 Preliminaries on molecular graph and GNNs

Consider a molecular graph denoted as G=(V,E), where V is the set of nodes representing the atoms in the molecule, and E⊆V×V is the set of edges indicating the chemical bonds. Each node $v_{i}\in V$ and edge $e_{ij}\in E$ is associated with a feature vector $x_{i}\in R^{d}$ and $e_{ij}\in R^{d^{\prime }}$, respectively. These vectors form the feature matrices $X\in R^{|V|\times d}$ and $E\in R^{|E|\times d^{\prime }}$, respectively.

GNNs are one of the neural encoder architectures specially designed for modeling graph data (Xia *et al.* 2023b). GNNs leverage the structural information of the graph to learn a embedding representation $h_{v_{i}}$ for each node $v_{i}\in V$, and the entire graph’s representation $h_{G}$. Typically, GNNs comprises multiple propagation layers and transformation layers. GNNs work by aggregating the features of neighboring nodes and edges, effectively merging the topological structure (Zhou *et al.* 2020). In the process, the contextual representation of each node can be updated. Formally, this is represented as:

(1) $$\begin{array}{c} h_{v_{i}}^{\left(k\right)}=\text{UPDATE}(h_{v_{i}}^{\left(k-1\right)},\text{AGGREGATE}\\ (\{(h_{v_{i}}^{\left(k-1\right)},h_{u}^{\left(k-1\right)},e_{uv}):u\in N(v)\})), \end{array}$$

where $h_{v_{i}}^{\left(k\right)}$ is the embedding representation of node *v* at the *k*th iteration, $e_{uv}$ denotes the feature vector of the edge between nodes *u* and *v*, N(v) corresponds to the neighbor nodes of *v*, and we initialize $h_{v_{i}}^{\left(0\right)}=x_{i}$. Subsequently, a graph-level representation can be obtained by performing a pooling operation over all the nodes (Mesquita *et al.* 2020):

(2) $$\begin{array}{c} h_{G}=\text{READOUT}\left(\left\{h_{v_{i}}^{\left(L\right)}|v\in V\right\}\right). \end{array}$$

### 2.2 Contrastive learning based on attribute masking

Hu *et al.* (2020) proposed the attribute masking method (AttrMask), which allows GNNs to learn the distribution of attributes on the graph structure by masking a portion of node/edge attributes. Specifically, for the input molecular graph, similar to the masked language model in the field of natural language processing (Devlin *et al.* 2019), we initially mask a certain proportion of node/edge attributes randomly and replace them with specific indicators to obtain a new masked graph $G_{m}$. The node attributes in the molecular graph include atomic types and chiral labels, while the edge attributes include the type and direction of the chemical bonds. We then train the model to learn the embeddings of the masked parts and finally apply a linear model to the embeddings to reconstruct the node/edge attributes. The reconstruction loss of node-level AttrMask is

(3) $$\begin{array}{c} L_{\text{Mask}}=-\sum_{G\in D}\sum_{v_{i}\in m(V)}\;\text{log}\;p(x_{v_{i}}|G_{m}), \end{array}$$

where D denotes the datasets, m(V) are the masked nodes from the node set V. AttrMask predicts the masked attributes based on the context, enabling the GNNs to capture simple chemical rules (Xia *et al.* 2023b). In order to further capture the molecular graph level semantics, we design a graph-level contrastive learning task based on attribute masking. By inputting the masked graph $G^{m}$ into the GNNs, we generate a graph embedding $h_{G_{m}}$. We consider it and the output of the original molecular graph $h_{G}$ as a positive pair, which can be formulated as

(4) $$\begin{array}{c} h_{G}=f(G;\theta ),h_{G_{m}}=f(G_{m};\theta ). \end{array}$$

Introducing a projection head between the feature representation and the contrastive loss can significantly optimize the quality of the learned features Chen *et al.* (2020). Therefore, we use a two-layer perceptron (MLP) to generate the final feature representation:

(5) $$\begin{array}{c} z_{G}=g(G),z_{G_{m}}=g(G_{m}). \end{array}$$

The contrastive learning loss is designed using the normalized temperature-scaled cross entropy loss (NT-Xent) (Chen *et al.* 2020):

(6) $$\begin{array}{c} L_{\text{CL}1}=-\sum_{G\in D}\;\text{log}\;\frac{\;\text{exp}(\text{sim}(z_{G},z_{G_{m}})/\tau )}{\sum_{G^{\prime }\in B,G^{\prime }\neq G}\;\text{exp}\;(\text{sim}(z_{G},z_{G^{\prime }})/\tau )}, \end{array}$$

where B represents the sampled batch including G, τ is the temperature hyper-parameter, and sim(·,·) denotes the cosine similarity. By combining the reconstruction loss and contrastive loss of AttrMask, the GNNs can simultaneously capture node-level and graph-level molecular semantics. We refer to this as the attribute-masking graph contrastive learning module, MaskGCL, whose hybrid loss is

(7) $$\begin{array}{c} L_{\text{MaskGCL}}=\alpha \;L_{\text{Mask}}+(1-\alpha )\;L_{\text{CL}1}, \end{array}$$

where α is the trade-off hyperparameter.

### 2.3 Contrastive learning based on parameter perturbation

MaskGCL achieves molecular contrastive learning through simple augmentation operations, eliminating the need for laborious manual trial and errors. However, more and more research suggests that simple enhancements to molecular structures may result in a loss of semantics (Xia *et al.* 2022, Gong *et al.* 2023). In order to better preserve the semantic information of the original molecular graph and to learn feature-level information, we design a graph contrastive learning method based on parameter perturbation, PPGCL.

Xia *et al.* (2022) propose directly perturbing model parameters using Gaussian noise. However, Gong *et al.* (2023) point out that the influence of model parameters on encoding semantics is highly complex, and meaningless noise may damage semantic representations in encoding. Our experiments also confirmed this observation by revealing negative transfer effects (the effect after pre-training is actually worse than the unpretrained model) caused by SimGRACE. Hence, the design of perturbations is crucial.

The alignment metric, which suggest that positive examples should remain closely bound in the embedding space, is frequently employed to assess the quality of representations derived through contrastive learning (Wang and Isola 2020). Meanwhile, research in domain adaptation (Ganin and Lempitsky 2015) indicates that using gradient reversal layers in backpropagation training can align the feature distributions in two domains. Therefore, we design a parameter perturbation based on gradient compensation using the contrastive loss gradient $\nabla_{\theta }L_{\text{CL}1}$ from the MaskGCL stage. This is mathematically described as

(8) $$\begin{array}{c} \theta_{l}^{\prime }=\theta_{l}\;+\;\eta \cdot \nabla_{\theta_{l}}L_{\text{CL}1} \end{array}$$

where $\theta_{l}$ and $\theta_{l}^{\prime }$ represent the weight tensors of the *l*th layer of the GNN encoder and its corresponding perturbed version. η is a hyperparameter used to control the magnitude of the perturbation. Our method differs from SimGRACE by capitalizing on the contrastive learning gradient from the previous step.

We introduce the perturbation to the parameters θ of the GNN encoder f(·;θ), which has been trained in the MaskGCL module. This process yields new graph-level representations $h_{G}^{\prime }$ without altering the input of the molecular graph:

(9) $$\begin{array}{c} h_{G}^{\prime }=f(G;\theta^{\prime }),z_{G}^{\prime }=z(h_{G}^{\prime }). \end{array}$$

Following the incorporation of gradient compensation, our experiments demonstrate that GNNs not only achieve accurate representations of the molecular graph but also maintain closer proximity between positive samples. This improvement contributes to enhancing the performance of subsequent contrastive learning. Similar to the MaskGCL module, we consider the features obtained from the encoder with parameter perturbation and the original features as a positive pair for contrastive learning:

(10) $$\begin{array}{c} L_{\text{PPGCL}}=-\sum_{G\in D}\;\text{log}\;\frac{\;\text{exp}(\text{sim}(z_{G},z_{G}^{\prime })/\tau )}{\sum_{G^{\prime }\in B,G^{\prime }\neq G}\;\text{exp}\;(\text{sim}(z_{G},z_{G^{\prime }})/\tau )}. \end{array}$$

### 2.4 Multi-level graph contrastive learning

**Figure 1.**
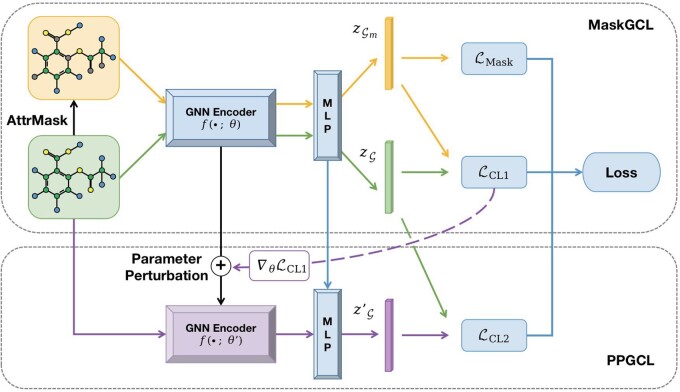
Overview of our molecular pre-training framework, MoleMCL. In the MaskGCL module (upper half), two concurrent training objectives are employed: attribute masking for reconstruction training and contrastive learning across two views. The PPGCL module (lower half) leverages the contrastive learning loss from MaskGCL to introduce gradient-based perturbations to the GNN encoder parameters, thereby generating new positive samples for further contrastive learning.

Finally, as shown in Fig. 1, our training process involves the joint optimization of the reconstruction and contrastive losses from MaskGCL, along with the contrastive loss in PPGCL:

(11) $$\begin{array}{c} L=L_{\text{MaskGCL}}+L_{\text{PPGCL}}. \end{array}$$

It’s crucial to note that this isn’t a simple concatenation of two modules. The computation of gradients in PPGCL relies on the contrastive learning in MaskGCL. Traditional gradient-based techniques are not directly applicable to molecular tasks due to the lack of the abundant labels needed for gradient computation in molecular datasets. Therefore, we employ unsupervised contrastive learning to provide computable gradients for parameter perturbation.

## 3 Experiments

In this section, we present experiments conducted on the task of molecular property prediction to substantiate the effectiveness of MoleMCL. We delineate the experimental datasets and associated settings, compare the performance of MoleMCL with SOTA methods in the field of graph pre-training, and perform ablation studies to justify our approach. Furthermore, we incorporate a molecular retrieval experiment to assess MoleMCL’s capability to generate chemically significant representations.

### 3.1 Experimental setup

In the pre-training stage, following the previous works of Hu *et al.* (2020) and Xia *et al.* (2023a), we conduct self-supervised pre-training on 2 million unlabeled molecules sampled from the ZINC15 database (Sterling and Irwin 2015). For the downstream molecular property prediction tasks, we use eight binary classification datasets included in the commonly used molecular property prediction benchmark, MoleculeNet (Wu *et al.* 2018). Notably, to better assess the model’s out-of-distribution generalization ability, we employ *scaffold splitting* (Ramsundar *et al.* 2019). This means that molecules are sorted and divided based on their structures, ensuring that the structures of the molecules in the training and test sets differ to the greatest possible extent.

In our experiments, we primarily utilize graph isomorphism network (GIN) (Xu *et al.* 2019) as our neural network architecture. The GIN model we use comprises five layers, whose hidden dimension is 300. We also elect to utilize mean pooling as the readout function for our GNNs. Such settings are designed to ensure a fair comparison of our experiments with earlier studies (Hu *et al.* 2020, Xia *et al.* 2023a). First, we pre-train the GNNs for 100 epochs with a batch size of 256 and a learning rate of 0.001. Then, during the fine-tuning phase, we train for 100 epochs with a batch size of 32. For downstream experiments, we used 10 random seeds, divided the training/validation/testing set by 8:1:1, and reported the test performance of the best validation round using the ROC-AUC and the validation early stopping protocol. Due to the adoption of different evaluation protocols in previous studies, the results of certain baselines may differ from their original papers. Both α (trade-off hyperparameter) and μ (gradient weight hyperparameter) are selected as the optimal values from 0.3, 0.5, 0.7 and 2, 4, 6, 8, 10, respectively, based on their best performance on the validation set, which can be seen in Supplementary Section 2.

### 3.2 Comparison with baseline methods

We select a variety of GNN pre-training baselines, which include the latest state-of-the-art methods for molecular pre-training: EdgePred (Hamilton *et al.* 2017), InfoMax (Veličković *et al.* 2019), ContextPred (Hu *et al.* 2020), AttrMask (Hu *et al.* 2020), GraphCL (You *et al.* 2020), GraphMAE (Hou *et al.* 2022), redGraphMVP (Liu *et al.* 2022), JOAO (You *et al.* 2021), JOAOv2 (You *et al.* 2021), SimGRACE (Xia *et al.* 2022), and Mole-BERT (Xia *et al.* 2023a). In addition, we also consider a new graph contrast learning module, TMCL, proposed in Mole-BERT. To ensure a fair comparison, we directly adopt the trained models suggested by the first seven works. For Mole-BERT and TMCL, we use their publicly available source code and employ the training parameters recommended in their papers.

We present the results of molecular property prediction tasks in Table 1. These results express the average (and standard deviation) of the ROC-AUC for 10 random seeds under the condition of molecular scaffold splitting. The best and second-best results are highlighted in bold and underlined respectively. Additionally, “No Pretrain” means that the model was trained from scratch.

**Table 1.** Results for classification tasks on AUC-ROC (higher is better) with the scaffold split.

Dataset	Tox21	ToxCast	Sider	clintox	MUV	HIV	BBBP	Bace	Avg
No. of molecules	7831	8575	1427	1478	93 087	41 127	2039	1513	–
No. of binary prediction tasks	12	617	27	2	17	1	1	1	–
No pretrain	73.9 (0.8)	61.9 (0.6)	55.8 (3.0)	55.6 (4.5)	70.1 (2.7)	74.3 (2.0)	64.4 (2.0)	65.9 (5.0)	65.2
EdgePred	75.5 (0.5)	62.9 (1.0)	59.4 (0.8)	64.1 (3.8)	72.9 (2.5)	76.1 (0.7)	64.3 (2.2)	78.6 (1.2)	69.2
InfoMax	75.1 (0.5)	62.7 (0.4)	58.0 (0.8)	69.3 (2.3)	74.4 (1.1)	76.1 (0.7)	67.6 (1.0)	75.5 (1.7)	69.8
ContextPred	75.0 (0.5)	63.5 (0.2)	59.6 (0.7)	65.8 (4.3)	75.2 (1.7)	76.3 (1.0)	66.8 (1.9)	77.9 (1.2)	70.0
AttrMask	76.0 (0.3)	63.7 (0.2)	59.3 (0.9)	71.1 (3.0)	72.4 (1.1)	76.9 (1.0)	64.0 (2.9)	78.8 (0.7)	70.3
GraphCL	74.2 (0.5)	62.8 (0.4)	59.5 (0.6)	70.1 (7.8)	71.9 (2.7)	77.3 (1.1)	68.9 (1.3)	74.4 (2.5)	69.9
GraphMAE	73.6 (2.2)	62.9 (1.7)	59.7 (0.8)	73.1 (5.0)	76.2 (1.8)	76.3 (1.1)	**69.0** (2.0)	**81.3** (1.4)	71.5
GraphMVP	75.4 (0.5)	63.5 (0.5)	59.3 (1.1)	76.1 (4.8)	74.4 (2.2)	76.1 (1.7)	67.9 (2.0)	78.6 (3.5)	71.4
JOAO	74.7 (0.2)	63.6 (0.3)	60.6 (1.1)	78.1 (3.9)	74.2 (1.6)	76.6 (1.0)	68.0 (1.6)	77.3 (1.3)	71.6
JOAOv2	74.9 (0.7)	63.5 (0.3)	**61.1** (0.7)	78.6 (3.3)	75.3 (1.2)	76.9 (0.8)	68.0 (1.0)	75.3 (3.7)	71.7
SimGRACE	73.8 (0.4)	61.7 (0.3)	59.2 (0.6)	63.4 (2.3)	70.6 (1.5)	74.3 (0.9)	68.4 (0.8)	75.2 (0.6)	68.3
Mole-BERT	75.3 (0.6)	**63.9** (1.0)	59.5 (1.1)	77.8 (1.6)	75.4 (1.3)	77.6 (1.1)	67.4 (2.2)	78.9 (1.8)	72.0
TMCL	75.6 (0.5)	63.5 (0.4)	59.1 (1.0)	72.6 (2.7)	74.2 (3.0)	76.5 (1.4)	64.1 (2.4)	77.1 (2.4)	70.3
MoleMCL	**76.2** (0.6)	63.7 (0.2)	59.0 (0.8)	**80.2** (1.6)	**78.2** (1.0)	**79.6** (0.5)	67.8 (3.5)	80.3 (0.8)	**73.1**

We can observe that, under the same experimental setup, MoleMCL is able to match or even exceed the performance of previous pre-training strategies. More specifically, MoleMCL achieves SOTA performance on four out of eight datasets, with an improvement of up to 7.8% over models without pre-training, and a 1.1% improvement over the current best-performing method, Mole-BERT. Despite the complex two-step pre-training required by MoleBERT, it implies that it needs a longer training time and more computational resources.

Next, to demonstrate that MoleMCL can serve as foundational pre-training tasks to optimize molecular modeling, we incorporated them as preliminary steps in supervised training, with results outlined in the Table 2. The supervised pre-training approach adheres to prior works (Hu *et al.* 2020), drawing data from the pre-processed ChEMBL dataset (Gaulton *et al.* 2012), some of whose labels originate from biochemical analyses. It can be observed that, even in the presence of labeled supervision, MoleMCL still yields training gains.

**Table 2.** Performance comparison of MoleMCL as a pre-training step for supervised learning.

Dataset	Tox21	ToxCast	Sider	clintox	MUV	HIV	BBBP	Bace	Avg.
No pretrain	76.4 (0.4)	64.1 (0.3)	61.6 (0.7)	55.9 (2.1)	77.5 (2.5)	74.6 (0.8)	68.5 (1.3)	77.7 (0.6)	69.5
EdgePred	78.3 (0.5)	**66.0** (0.4)	62.5 (0.7)	68.5 (2.9)	78.4 (1.9)	76.3 (0.9)	65.5 (0.9)	80.2 (1.2)	72.0
InfoMax	77.5 (0.4)	64.8 (0.5)	60.5 (0.6)	68.0 (2.5)	77.6 (3.3)	76.9 (0.8)	68.1 (2.4)	80.5 (1.2)	71.8
ContextPred	77.9 (0.4)	65.5 (0.7)	62.3 (0.5)	72.0 (2.3)	79.1 (0.8)	77.0 (2.2)	68.7 (1.3)	**83.9** (0.6)	73.3
AttrMask	77.8 (0.3)	65.1 (0.3)	63.1 (0.8)	72.7 (5.0)	79.6 (1.8)	76.1 (0.9)	66.4 (3.0)	81.5 (1.0)	72.8
Mole-BERT	78.2 (0.4)	65.1 (0.3)	62.2 (0.5)	72.1 (5.1)	78.0 (2.8)	76.4 (1.1)	68.8 (3.1)	82.4 (1.6)	72.9
MoleMCL	**78.6** (0.4)	65.2 (0.3)	**63.7** (0.4)	**74.0** (3.9)	**81.8** (1.3)	**77.6** (0.6)	**71.6** (3.0)	81.1 (3.0)	**74.2**

### 3.3 Ablation studies

We conducted ablation experiments to demonstrate the effectiveness of our model design. We compared SimGRACE (Contrastive Learning based on Gaussian Noise Parameter Perturbation), MaskMCL (Contrastive Learning based on AttrMask), PPGCL (Contrastive Learning based on Parameter Perturbation), MaskGCL + SimGRACE (trained with both contrastive losses) against our model. Note that the variant MaskGCL + SimGRACE adopts the same architecture and parameter α as MoleMCL, the only difference being that its perturbation to parameters is simply Gaussian noise. The results are shown in Table 3. From the experimental results, the following insights can be drawn:

**Table 3.** Ablation studies of MoleMCL.

Dataset	Tox21	ToxCast	Sider	clintox	MUV	HIV	BBBP	Bace	Avg.
No pretrain	73.9 (0.8)	61.9 (0.6)	55.8 (3.0)	55.6 (4.5)	70.1 (2.7)	74.3 (2.0)	64.4 (2.0)	65.9 (5.0)	65.2
SimGRACE	73.8 (0.4)	61.7 (0.3)	**59.2** (0.6)	63.4 (2.3)	70.6 (1.5)	74.3 (0.9)	**68.4** (0.8)	75.2 (0.6)	68.3
MaskGCL	75.0 (0.3)	63.5 (0.6)	59.0 (1.0)	70.2 (3.0)	75.5 (0.8)	76.7 (1.4)	65.2 (2.5)	78.5 (2.5)	70.4
PPGCL	74.6 (0.8)	63.1 (0.7)	57.2 (1.3)	62.6 (3.4)	74.4 (2.3)	76.7 (1.0)	67.2 (2.5)	72.5 (3.5)	68.5
MaskGCL + SimGRACE	75.6 (0.4)	62.7 (0.9)	58.4 (0.6)	58.9 (2.1)	74.5 (1.0)	76.5 (0.9)	61.9 (1.9)	76.8 (1.1)	68.2
MoleMCL	**76.2** (0.6)	**63.7** (0.2)	59.0 (0.8)	**80.2** (1.6)	**78.2** (1.0)	**79.6** (0.5)	67.8 (3.5)	**80.3** (0.8)	**73.1**

1. Our proposed MaskGCL, PPGCL, and MoleMCL show improvements compared to models without pre-training, indicating effective pre-training strategies. Moreover, MoleMCL exhibits improvements of 1.5% and 4.6% compared to MaskGCL and PPGCL, respectively, demonstrating that combining two enhancement methods can effectively enhance model performance.
2. SimGRACE and its variant, MaskMCL + SimGRACE, exhibited negative transfer on certain datasets, indicating that simple Gaussian noise perturbations may disrupt the model’s semantics, leading to performance deterioration. Adding SimGRACE to the model resulted in decreased performance compared to using MaskGCL alone, suggesting the need for careful enhancement method design. Our proposed PPGCL, perturbing encoder parameters based on the previous contrastive loss gradient, effectively integrates new feature semantics while preserving MaskGCL’s learned structural information, improving model performance.

Lastly, we examined the impact of different GNNs backbones. As shown in Table 4, we evaluated the pre-training benefits of MaskGCL and MoleMCL across three popular GNN architectures including GraphSage (Hamilton *et al.* 2017), GCN (Kipf and Welling 2017), and GIN (Xu *et al.* 2019). The results indicate that, compared to training various GNNs from scratch, both strategies achieved certain improvements. Moreover, notably, MoleMCL consistently enhances the performance of MaskGCL across all backbones.

**Table 4.** The influence of GNNs backbone.

Model	GraphSage	GCN	GIN
No pretrain	66.0	67.2	65.2
MaskGCL	69.4	69.2	70.4
MoleMCL	70.8	70.2	73.0
Relative gain	+4.8%	+3.0%	+7.8%

### 3.4 Molecular retrieval

**Figure 2.**
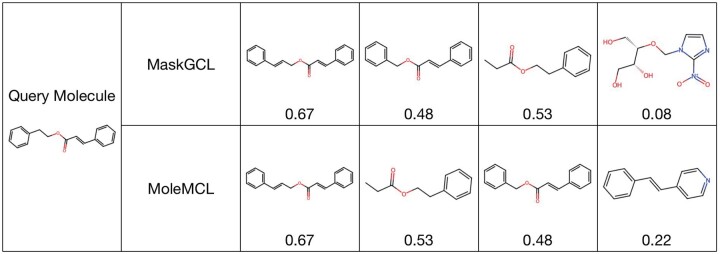
The query molecule alongside the four closest molecules with the extracted representations. The Tanimoto similarity scores, displayed below each molecule, quantify the chemical resemblance to the query molecule.

The molecular retrieval experiment aims to ascertain the capacity of MoleMCL to acquire chemically significant representations for practical application. Implemented on the ToxCast dataset, this experiment involves calculating the cosine similarities between the representations of all test set molecules and a designated query molecule. Figure 2 illustrates the top four molecules that exhibit the highest cosine similarity to the query molecule, in addition to their Tanimoto similarity scores. The results demonstrate that the representations derived by MoleMCL align with molecular fingerprint similarities, whereas those obtained by MaskMCL do not accurately capture genuine chemical resemblances. This substantiates the effectiveness of our tailored gradient compensation graph contrastive learning approach, affirming its necessity and efficacy in enhancing molecular feature information. More ablations and results can be seen in Supplementary Section 3.

## 4 Conclusion

In this article, we uncover the limitations of existing graph augmentation techniques and MPMs. To address the negative transfer issue in SimGRACE caused by the randomness of Gaussian noise, we devised a perturbation scheme based on gradient compensation. Moreover, recognizing that current molecular contrastive learning methods focus solely on either structural or feature semantics, we integrated attribute masking with parameter perturbation-based contrastive learning, leading to the multi-level contrastive learning molecular pre-training framework MoleMCL. Experiments demonstrate that MoleMCL achieves outstanding performance on molecular property prediction tasks. Through experimentation, we also demonstrated that our proposed contrastive learning strategy can serve as a universal pre-training task. We hope this work will further the understanding and exploration of molecules and also pave new avenues for self-supervised learning on graphs. Future endeavors will contemplate extending the gradient compensation strategy to other tasks to better tackle out-of-distribution generalization challenges.

## Supplementary Material

btae164_Supplementary_Data
