# Supplementary material for: MoleMCL: a multi-level contrastive learning framework for molecular pre-training
Source: Bioinformatics. 2024 Mar 26;40(4):btae164. doi: 10.1093/bioinformatics/btae164 (PMC11001485; doi:10.1093/bioinformatics/btae164)
Supplement: btae164_Supplementary_Data [file btae164_supplementary_data.pdf]

Supplementary Materials for PAPER: MoleMCL: A Multi-level Contrastive Learning Framework for Molecular Pre-training

1 Related Works

1.1 Pre-training on Molecules

Inspired by the success of NLP, Molecular Pre-trained Models (MPMs) have been introduced to learn general molecular representation. Existing pre-training strategies can be broadly categorized into supervised pre-training and self-supervised pre-training. The study by Hu et al. (2020) proposed a graph-level supervised task, which, however, is constrained by the limited number of molecular labels and exhibits negative transfer in downstream tasks. In comparison, self-supervised pre-training appears more suitable for molecules. Initially, researchers adopted sequence-based pre-training approaches for string-based molecular data, such as SMILES transformer (Honda et al., 2019), ChemBERTa (Chithrananda et al., 2020), and MolGPT (Bagal et al., 2022). Given that molecules can be represented as 2D graphs in which each node and edge can possess associated features, several models have emerged that leverage graph topology for molecular representation learning. As a pioneering effort, Hu et al. (2020) introduced strategies of attribute masking and predicting contextual subgraphs. With the incorporation of contrastive learning, GraphCL (You et al., 2020) and its variants(Fang et al., 2022; Pinheiro et al., 2022; Sun et al., 2021; Suresh et al., 2021; Wang et al., 2021, 2022; Xu et al., 2021) aim to learn molecular representations by drawing augmented molecules closer to their anchor counterparts. Meanwhile, InfoGraph (Sun et al., 2020) and MVGRL (Hassani and Ahmadi, 2020) employ contrastive learning by maximizing mutual information. In the latest work, Mole-BERT (Xia et al., 2023) introduced a novel atomic encoding method, achieving optimal performance through node-level masking and graph-level contrastive tasks. The 3D geometric structure of a molecule can encode conformational information, from which many molecular properties can be inferred. Zaidi et al. (2023) introduced noise into atomic coordinates in 3D ensembles to learn molecular force fields. Based on the 2D graph structure of molecules, our proposed framework employs multi-level contrastive learning, capturing both structural information and semantic features of molecules, unveiling a new avenue for molecular modeling.

1.2 Graph Contrastive Learning

Based on the contrastive granularity, we can categorize graph contrastive learning into two classes: cross-level contrast and instance discrimination. The former contrasts between the

representations of a graph and its substructures of different granularities. In InfoGraph (Sun et al., 2020), substructures are nodes, edges, and triangles, while in GCC (Qiu et al., 2020), they are sampled subgraphs. A follow-up study, MVGRL (Hassani and Ahmadi, 2020) , performs node diffusion to generate an augmented molecular graph and contrasts the atom representations of one view with the molecular representations of another. The latter class constructs positive samples for contrastive learning by augmenting graph data. Early works like GRACE (Zhou et al., 2020) and GraphCL (You et al., 2020) introduced random perturbations to the graph structure to construct contrastive views. However, it's pointed out that for structurally sensitive graph data like molecules, minor perturbations might lead to significant property alterations. Simultaneously, to overcome the manual trial-and-error limitation of GraphCL, JOAO (You et al., 2020) employs a unified dual-level optimization framework to automatically select augmentations for specific graph data, with other similar adaptive augmentation methods being GCA (Zhu et al., 2021) and AD-GCL (Suresh et al., 2021). MoCL (Sun et al., 2021), aiming to preserve the semantic value of molecular graphs, incorporates expensive domain knowledge. Moreover, there are feature-based approaches. GASSL (Yang et al., 2021) introduces perturbations on init feature and hidden layer, while SimGRACE (Xia et al., 2022) applies Gaussian noise perturbations to the GNN encoder parameters. However, in our experiments, we found that SimGRACE exhibits severe negative transfer on molecular data, indicating that merely adding noise doesn't guarantee consistent effectiveness. Therefore, building upon SimGRACE, we propose a more scientific augmentation method by perturbing parameters using gradient compensation.

2 The algorithmic process of MoleMCL

The pseudocode of MoleMCL is shown in Algorithm 1.

Algorithm 1 The pre-training procedure of MoleMCL

Input: Dataset $\mathcal{D} = \{\mathcal{G}_i\}_{i=1}^{N}$; The masking ratio of attributes r_m;
Output: Trained parameters θ.

Initialize the graph neural network parameters θ.
for each batch $\mathcal{B}$ in $\mathcal{D}$ **do**
    Generate masked graphs $\mathcal{G}_m$.
    Compute the feature representations $z_{\mathcal{G}}$ and $z_{\mathcal{G}_m}$ for $\mathcal{G}$ and $\mathcal{G}_m$.
    Compute $\mathcal{L}_{\text{Mask}}$ and $\mathcal{L}_{\text{CL1}}$ to get $\mathcal{L}_{\text{MaskGCL}}$.
    Perturb the parameters of the GNN encoder using gradients from $\mathcal{L}_{\text{CL1}}$.
    Generate new positive samples $z'_{\mathcal{G}}$ using the perturbed GNN encoder.
    Compute the contrastive loss $\mathcal{L}_{\text{PPGCL}}$ between $z_{\mathcal{G}}$ and $z'_{\mathcal{G}}$.
    Update the parameters θ based on the combined loss $\mathcal{L} = \mathcal{L}_{\text{MaskGCL}} + \mathcal{L}_{\text{PPGCL}}$.
end for
return trained parameters θ.

Table 1: The performance with various α on 8 MoleculeNet datasets.

trade-off hyperparameter α	0.3	0.5	0.7
MoleMCL	70.5	73.1	71.1

Table 2: The performance with various gradient weight μ on 8 MoleculeNet datasets.

gradient weight μ	2	4	6	8	10
MoleMCL	70.0	71.8	73.1	70.7	71.6

3 The Influence of the hyperparameters

In the context of two given hyperparameters, namely the Trade-off Hyperparameter (α) and the Gradient Weight (μ), we conducted systematic performance experiments.

α is used to adjust the weighting of the impact between the node-level reconstruction task and the graph-level contrastive learning task in the MaskGCL module. It can be observed from the table 1 that when α increases from 0.3 to 0.5, the performance of the MoleMCL model significantly improves. However, as α further increases to 0.7, the performance experiences a decline. Therefore, selecting an appropriate α value is crucial, requiring a balance between the two tasks.

μ controls the magnitude of parameter perturbation, where a larger μ corresponds to a greater influence of gradients on parameter perturbation. The μ hyperparameter in Table 2 regulates the impact of gradients on parameter perturbation, with larger μ values leading to more significant perturbation effects. As μ increases from 2 to 6, the MoleMCL model's performance shows an upward trend, followed by a slight decline after reaching 8. This suggests that a moderate μ value contributes to enhancing model performance, but beyond a certain threshold, increasing μ may lead to a decrease in model performance.

4 More Results of Molecule Retrieval

We present additional molecular retrieval results in Figure 1, where MaskGCL+SimGRACE denotes training with a combination of two contrastive losses. It is noteworthy that the retrieval results of MaskGCL and MaskGCL+SimGRACE are not entirely satisfactory. Although MaskGCL+SimGRACE can identify more similar molecules compared to MaskGCL, it fails to categorize molecules in a chemically meaningful order.Hence, while the combination of methods at the feature level is crucial, it still requires careful design of appropriate enhancement strategies. Our proposed PPGCL, combined with MaskGCL, achieves accurate molecular retrieval. It not only identifies molecules with the highest similarity but also arranges them in a sequence that aligns with practical chemical significance.

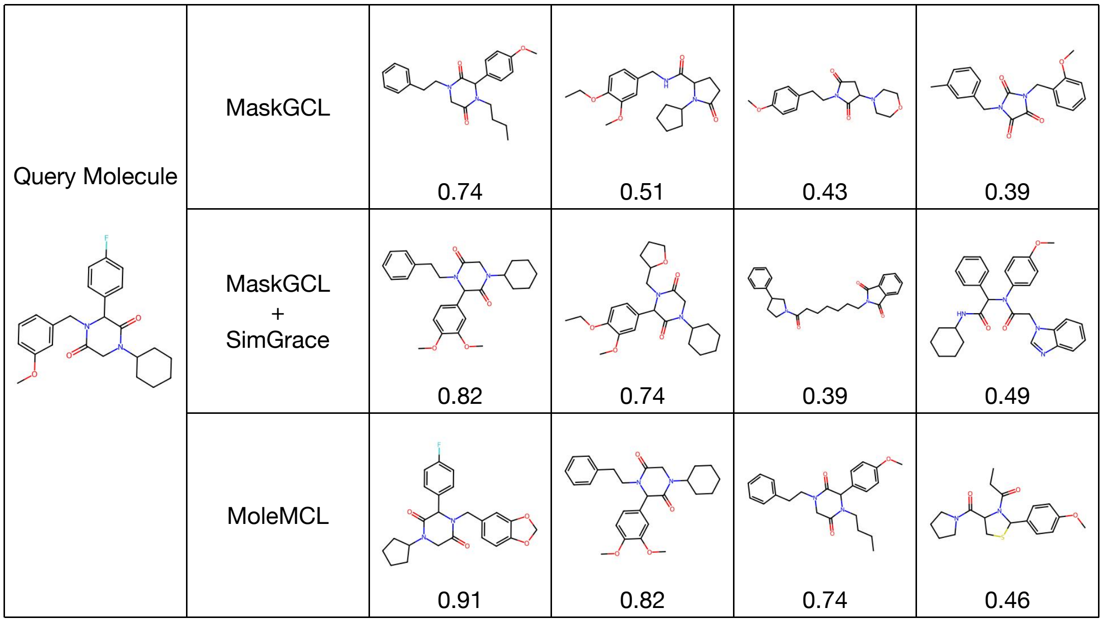

Figure 1: The query molecule alongside the 4 closest molecules with the extracted representations. The Tanimoto similarity scores, displayed below each molecule, quantify the chemical resemblance to the query molecule.

Table 3: Training times of pre-trained models

**Model**	JOAO	JOAOv2	Mole-BERT	MoleMCL
**Training Time (hours)**	32	30	29	21

5 Analysis of Training Times

Table 3 compares the training times of four pre-trained models conducted on the NVIDIA A100 80GB GPU. Among them, JOAO and JOAOv2 are based on an adversarial training framework with automatic data augmentation selection during training. Mole-BERT requires a two-step training process, first generating a vocabulary through VQVAE and then conducting molecular representation pre-training. On the other hand, MoleMCL, our model, directly explores molecules through multi-level contrastive learning.

MoleMCL stands out with the shortest training time of 21 hours, showcasing its efficiency in learning molecular representations. In contrast, JOAOv2 and JOAO have longer training times of 30 and 32 hours, respectively, indicating higher computational demands due to their complex training frameworks.

References

V. Bagal, R. Aggarwal, P. K. Vinod, and U. D. Priyakumar. Molgpt: Molecular generation using a transformer-decoder model. *J. Chem. Inf. Model.*, 62(9):2064–2076, 2022.

S. Chithrananda, G. Grand, and B. Ramsundar. Chemberta: Large-scale self-supervised pretraining for molecular property prediction. *arXiv Preprint*, 2020. URL `https://arxiv.org/abs/2010.09885`.

Y. Fang, Q. Zhang, H. Yang, X. Zhuang, S. Deng, W. Zhang, M. Qin, Z. Chen, X. Fan, and H. Chen. Molecular contrastive learning with chemical element knowledge graph. In *AAAI*, pages 3968–3976. AAAI Press, 2022.

K. Hassani and A. H. K. Ahmadi. Contrastive multi-view representation learning on graphs. In *ICML*, volume 119 of *Proceedings of Machine Learning Research*, pages 4116–4126, 2020.

S. Honda, S. Shi, and H. R. Ueda. Smiles transformer: Pre-trained molecular fingerprint for low data drug discovery. *arXiv Preprint*, 2019. URL `https://arxiv.org/abs/1911.04738`.

W. Hu, B. Liu, J. Gomes, M. Zitnik, P. Liang, V. S. Pande, and J. Leskovec. Strategies for pre-training graph neural networks. In *ICLR*, 2020.

G. A. Pinheiro, J. L. F. D. Silva, and M. G. Quiles. SMICLR: contrastive learning on multiple molecular representations for semisupervised and unsupervised representation learning. *J. Chem. Inf. Model.*, 62(17):3948–3960, 2022.

J. Qiu, Q. Chen, Y. Dong, J. Zhang, H. Yang, M. Ding, K. Wang, and J. Tang. GCC: graph contrastive coding for graph neural network pre-training. In *KDD*, pages 1150–1160. ACM, 2020.

F. Sun, J. Hoffmann, V. Verma, and J. Tang. Infograph: Unsupervised and semi-supervised graph-level representation learning via mutual information maximization. In *ICLR*. OpenReview.net, 2020.

M. Sun, J. Xing, H. Wang, B. Chen, and J. Zhou. Mocl: Data-driven molecular fingerprint via knowledge-aware contrastive learning from molecular graph. In *KDD*, pages 3585–3594. ACM, 2021.

S. Suresh, P. Li, C. Hao, and J. Neville. Adversarial graph augmentation to improve graph contrastive learning. In *NeurIPS*, pages 15920–15933, 2021.

Y. Wang, Y. Min, E. Shao, and J. Wu. Molecular graph contrastive learning with parameterized explainable augmentations. In *BIBM*, pages 1558–1563. IEEE, 2021.

Y. Wang, R. Magar, C. Liang, and A. B. Farimani. Improving molecular contrastive learning via faulty negative mitigation and decomposed fragment contrast. *J. Chem. Inf. Model.*, 62(11):2713–2725, 2022.

J. Xia, L. Wu, J. Chen, B. Hu, and S. Z. Li. Simgrace: A simple framework for graph contrastive learning without data augmentation. In *WWW*, pages 1070–1079, 2022.

J. Xia, C. Zhao, B. Hu, Z. Gao, C. Tan, Y. Liu, S. Li, and S. Z. Li. Mole-bert: Rethinking pre-training graph neural networks for molecules. In *ICLR*, 2023.

D. Xu, W. Cheng, D. Luo, H. Chen, and X. Zhang. Infogcl: Information-aware graph contrastive learning. In *NeurIPS*, pages 30414–30425, 2021.

L. Yang, L. Zhang, and W. Yang. Graph adversarial self-supervised learning. pages 14887–14899, 2021.

Y. You, T. Chen, Y. Sui, T. Chen, Z. Wang, and Y. Shen. Graph contrastive learning with augmentations. In *NeurIPS*, 2020.

S. Zaidi, M. Schaarschmidt, J. Martens, H. Kim, Y. W. Teh, A. Sanchez-Gonzalez, P. W. Battaglia, R. Pascanu, and J. Godwin. Pre-training via denoising for molecular property prediction. 2023.

J. Zhou, G. Cui, S. Hu, Z. Zhang, C. Yang, Z. Liu, L. Wang, C. Li, and M. Sun. Graph neural networks: A review of methods and applications. *AI Open*, 1:57–81, 2020.

Y. Zhu, Y. Xu, F. Yu, Q. Liu, S. Wu, and L. Wang. Graph contrastive learning with adaptive augmentation. In *WWW*, pages 2069–2080, 2021.
